# TiO_2_–Alginate–Chitosan-Based Composites for Skin Tissue Engineering Applications

**DOI:** 10.3390/gels10060358

**Published:** 2024-05-22

**Authors:** Emma Bobu, Kata Saszet, Zsejke-Réka Tóth, Emőke Páll, Tamás Gyulavári, Lucian Baia, Klara Magyari, Monica Baia

**Affiliations:** 1 Faculty of Physics, Doctoral School of Physics, Babeș-Bolyai University, M. Kogălniceanu 1, RO-400084 Cluj-Napoca, Romania; emma.bobu@ubbcluj.ro (E.B.); kata.saszet@ubbcluj.ro (K.S.); zsejke.toth@ubbcluj.ro (Z.-R.T.); 2 Nanostructured Materials and Bio-Nano-Interfaces Center, Interdisciplinary Research Institute on Bio-Nano-Sciences, Babeș-Bolyai University, Treboniu Laurian 42, RO-400271 Cluj-Napoca, Romania; lucian.baia@ubbcluj.ro; 3 Faculty of Veterinary Medicine, University of Agricultural Science and Veterinary Medicine, 400372 Cluj-Napoca, Romania; pallemoke@gmail.com; 4 Department of Applied and Environmental Chemistry, University of Szeged, Rerrich B. sqr. 1., 6720 Szeged, Hungary; gyulavarit@chem.u-szeged.hu; 5 Faculty of Physics, Babeș-Bolyai University, M. Kogălniceanu 1, RO-400084 Cluj-Napoca, Romania; 6 Institute of Research-Development-Innovation in Applied Natural Sciences, Babeș-Bolyai University, 400294 Cluj-Napoca, Romania; 7 INSPIRE Research Platform InfoBioNano4Health & Biomedical Imaging, Babeș Bolyai University, Arany Janos 11, 400084 Cluj-Napoca, Romania

**Keywords:** titanium dioxide, alginate, chitosan, hydrogel composites, hydroxyapatite layer, UV stability

## Abstract

The UV-B component of sunlight damages the DNA in skin cells, which can lead to skin cancer and premature aging. Therefore, it is necessary to use creams that also contain UV-active substances. Many sunscreens contain titanium dioxide due to its capacity to absorb UV-B wavelengths. In the present study, titan dioxide was introduced in alginate and chitosan–alginate hydrogel composites that are often involved as scaffold compositions in tissue engineering applications. Alginate and chitosan were chosen due to their important role in skin regeneration and skin protection. The composites were cross-linked with calcium ions and investigated using FT-IR, Raman, and UV–Vis spectroscopy. The stability of the obtained samples under solar irradiation for skin protection and regeneration was analyzed. Then, the hydrogel composites were assayed in vitro by immersing them in simulated body fluid and exposing them to solar simulator radiation for 10 min. The samples were found to be stable under solar light, and a thin apatite layer covered the surface of the sample with the two biopolymers and titanium dioxide. The in vitro cell viability assay suggested that the anatase phase in alginate and chitosan–alginate hydrogel composites have a positive impact.

## 1. Introduction

The skin covers and protects the whole body from bacteria or other pathogens that harm the organs. The skin is formed in three layers: the epidermis, dermis, and hypodermis, and there are plenty of cells that protect it and provide it with color and elasticity [1]. Sunlight is vital for the human body and is a mixture of waves, both visible and invisible, and only the short invisible rays, called ultraviolet (UV) rays, can be harmful to people. UV energy is divided into UV-A (315–400 nm), UV-B (280–315 nm), and UV-C (100–280 nm) [2], but only the UV-A and UV-B rays can reach the earth’s surface, UV-C being absorbed by the earth’s atmosphere. Without proper coverage, the skin can be destroyed by the UV rays from the sun [3]. More precisely, various studies have demonstrated that UV-A radiation produces reactive oxidative species (ROS) that damage the molecules, inducing skin cancer, whereas UV-B radiation causes sunburn and destroys collagen or vitamin A production in the skin [4]. To prevent all the negative effects of sun exposure, some proper materials can be used to protect and regenerate the skin. Many sunscreens use titanium dioxide (TiO_2_) due to its ability to reflect, scatter, or adsorb UV radiation. Thus, it works like a physical sun protection filter with good stability under UV irradiation, and it also usually has an anti-bacterial character. TiO_2_ is used in biomedicine, engineering, food packaging, and water treatment [5]. In 2021, the European Union banned TiO_2_ as a food additive (E171) in the EU due to uncertainties around possible inflammation and neurotoxicity. In the pharmaceutical industry, its use is still allowed, which is why we consider it very important to employ it for the preparation of biopolymer composites and to evaluate their structural and morphological stability in different environmental conditions. According to the literature, the preferred TiO_2_ particle sizes are in the nanoscale (for example, between 10 and 50 nm) because they can agglomerate to form larger particles with a reduced void between them, which increases the sun protection factor (SPF). In other words, it increases the efficacy of sunscreen creams against UV radiation, and TiO_2_ is more stable in formulations [6,7]. Other things that can influence the stability of TiO_2_ particles are the shape, coatings, and crystallinity [8]. Currently, the most commonly employed TiO_2_ crystalline phase is anatase [9,10], and, therefore, in our study, anatase was used and evaluated in the form of nanoparticles.

Alginate and chitosan are naturally derived polysaccharides that can play an important role in skin regeneration and skin protection. Alginate is a biopolymer found in brown algae and consists of 1–4 β–D–mannuronic acid (M) and 1–4 α–L–guluronic acid (G) monomers. The main role of the alginate dressing in skin regeneration is that it can adsorb wound fluid, resulting in gels that maintain a physiologically moist environment, thus minimizing bacterial infections at the wound site [11]. Their other advantages are excellent biocompatibility, anti-oxidative, anti-inflammatory properties, and non-toxic character [12]. Chitosan, a natural polysaccharide extracted from chitin, is a potential ingredient in cosmetic formulations for skin care due to its advantages like its bacteriostatic, fungistatic, and anti-static characteristics, moisture retention, and its good compatibility with other common cosmetic ingredients [13]. Chitosan consists of (1→4)-2-amino-2-deoxy-β-d-glucan [14].

The need for the early-stage screening and testing of materials with medical applications led to the development of in vitro assays decades ago [15,16]. The in vitro tests carried out in this study consisted of the immersion of the samples in simulated body fluid (SBF) and the irradiation of the materials with a solar simulator to replicate conditions similar to those in the body or the environment in which the materials might be placed. SBF is an inorganic solution with ion concentrations like human plasma [17], while the solar simulator is a device that mimics sunlight for laboratory testing [18].

The present study aims to prepare composites by introducing anatase crystalline-phased TiO_2_ in alginate and then in chitosan and alginate. The European Union acknowledged that TiO_2_ can be added in cosmetics or skin products up to a 25% concentration, with anything higher than that deemed certainly toxic [19]. Thus, in view of this, the percentage of TiO_2_ added to the biopolymers was set at 10%. All the characteristics of the samples were structurally and morphologically investigated, followed by in vitro evaluation. To understand the possible changes that occur in the interaction with the skin, different environmental conditions that may occur in contact with the skin were followed step by step. The hydrogel composites were immersed in SBF and exposed to solar irradiation in both dried and SBF-soaked forms. The purpose of these evaluations was to assess the composite’s stability in different environmental conditions, as well as to evaluate the effect of TiO_2_ in a natural hydrogel composite and its stability in a simulated body condition.

## 2. Results and Discussion

### 2.1. TiO_2_ Particle Characteristics

To determine the characteristics of the TiO_2_ particles, the synthesized TiO_2_ was evaluated structurally and morphologically. The XRD patterns showed an 81% crystallinity degree, and the most intensive reflections were for the anatase crystalline phase (see Figure S1). After calculating the particle sizes with the Scherrer equation [20], the mean size of the obtained nanocrystallites was found to be around 8 nm. TEM micrograph demonstrated that the TiO_2_ particles were spherical and polydisperse (see Figure S1b,c). The XRD data, together with Raman and UV-vis spectra, confirmed that only the anatase crystalline phase was present in the TiO_2_ (see Figures S1–S3). To verify the TiO_2_ particles’ stability under different physiological conditions, the samples were immersed in simulated body fluid (SBF) for 1 day and were exposed to solar irradiation, dry and SBF-soaked. The results indicated that the TiO_2_ particles were stable after exposure to a short period under solar irradiation, both dry and SBF-soaked, and after immersion in SBF for only one day, no visible structural change was evidenced, as revealed in the recorded XRD data (see Figure S4).

### 2.2. Evaluation of the TiO_2_–Alginate Composite Behavior

#### 2.2.1. Cross-Linking of the TiO_2_–Alginate Composites

The composites were investigated using XRD, FT-IR, and Raman spectroscopy as well as UV–Vis absorption spectroscopy. The diffractograms of the Alg and 10TiO_2_–Alg composites were very similar and proved the amorphous character of the samples (Figure S6).

**Figure 1:**
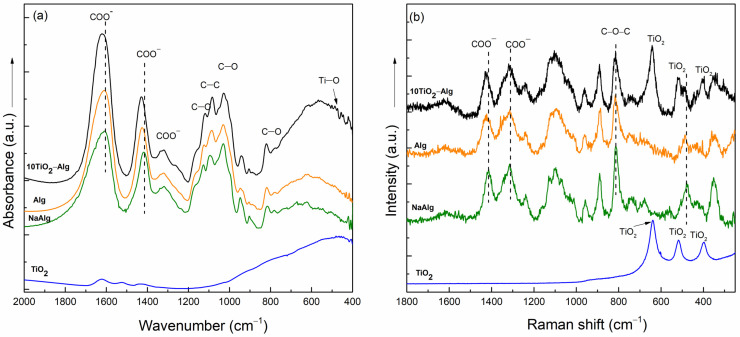
FT-IR (**a**) and Raman (**b**) spectra of TiO_2_ (blue line), NaAlg (green line), Alg (orange line), and the 10TiO_2_–Alg composite (black line).

The cross-linking between the TiO_2_ and alginate components into the hydrogel composite structure was demonstrated using FT-IR and Raman spectroscopy (Figure 1). The FT-IR spectrum of sodium alginate (Na-Alg) displayed the typical absorptions of anionic linear polysaccharides: the anti-symmetric and symmetric vibration bands of COO─ groups at 1610 and 1416 cm^−1^, the signal due to the C-O-C stretching vibration at 1120 cm^−1^, the absorption at 1090 cm^−1^ assigned to the C-C stretching vibrations, and the band attributed to the C-O stretching vibration from 1028 cm^−1^ [21,22]. The cross-linked alginate presents a shift in the absorption band due to the COO symmetric vibration from 1416 to 1425 cm^−1^ as a result of the cross-linking. The cross-linking between the alginate and TiO_2_ was also proven by the shift of the vibration bands of the COO^−^ groups from 1610 to 1623 cm^−1^. The large band noticed between 700 and 400 cm^−1^ in the FT-IR spectrum of the composite occurred as a consequence of the addition of TiO_2_.

The predominant Raman band present in the NaAlg spectrum at 1411 cm^−1^ and assigned to the stretching vibration of the COO^−^ groups shifted to 1425 cm^−1^ in the spectra of both cross-linked composites, Alg and 10TiO_2_–Alg. Another shift to higher wavenumbers can be seen in the Raman spectra of both composites (Alg and 10TiO_2_–Alg) for the band observed at 809 cm^−1^ in the NaAlg spectrum. These shifts are attributed to the interactions of alginate with the calcium ions from CaCl_2_ that were employed as cross-linking agents [23]. The presence of TiO_2_ in the 10TiO_2_–Alg composite structure is proven by the bands at 640, 514, and 398 cm^−1^ that are specific to TiO_2_ vibrations [24]. The results obtained from the Raman spectra analysis validate the ones derived from the FT-IR spectra, namely that the Ti^4+^ ion is also capable of cross-linking, thus forming the bond through the COO group [25].

**Figure 2:**
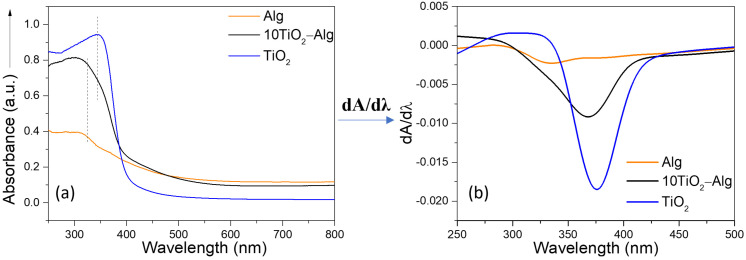
UV–Vis spectra (**a**) and first derivate spectra (**b**) of TiO_2_, Alg, and the 10TiO_2_–Alg composite.

In the UV–Vis spectrum of the pure Alg composite, a band at 320 nm and a shoulder around 340 nm were noticeable (Figure 2a). After applying the first derivative to the UV–Vis spectrum of the Alg sample, the minimum at 386 nm became more evident (see Figure S5). In the composite spectrum (Figure 2a,b), the typical bands for alginate and the typical threshold for anatase could be observed. After applying the first derivative to the samples’ spectrum (Figure 2b) and the deconvoluted spectrum (see Figure S5a), we noticed two different minimum values, one corresponding to alginate (324 nm) and the other one corresponding to anatase (367 nm). Thereby, it could be concluded that the hydrogel composite component linking was successful.

#### 2.2.2. In Vitro Assays of the TiO_2_–Alginate Composites

The hydrogel composites immersed in SBF and those that were dried and SBF-soaked after solar irradiation were further investigated using XRD, FT-IR, and Raman spectroscopy, and the surface changes were followed by SEM. SBF was used to simulate the biological environment, knowing that the concentration of ions in human plasma can influence the behavior of the samples by the appearance of the toxic compounds [26], and in the SBF, the ion concentrations are close to those in human plasma.

**Figure 3:**
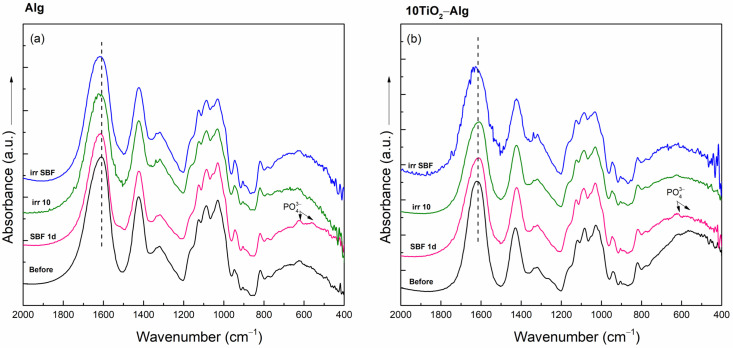
FT-IR spectra of the Alg (**a**) and 10TiO_2_–Alg (**b**) composites before treatment, after immersion in SBF for 1 day (SBF 1d), and after the solar irradiation of the dry (irr 10) and SBF-soaked samples (irr SBF) for 10 min.

The diffractograms of the Alg and 10TiO_2_–Alg composites after immersion in SBF for 1 day, as well as those recorded after solar irradiation of the dried samples and of the SBF-soaked samples for 10 min, were very similar; the amorphous characteristic of the samples is shown in Figure S6. So, neither the influence of SBF immersion nor that of the solar irradiation on the sample structure could be traced from the XRD analysis. The FT-IR spectra of the Alg and 10TiO_2_–Alg composites immersed in SBF for one day presented absorption bands at 604 and 564 cm^−1^ due to the apatite layer formation in the samples’ surface (Figure 3) [26]. After the solar irradiation of the dried and SBF-soaked Alg samples, small changes appeared in the FT-IR spectra: the carboxylic band at 1610 cm^−1^ shifted to 1620 cm^−1^ (Figure 3a). This indicates minor changes in the carboxylic group of Alg under solar irradiation. Similar shifts appeared in the spectrum of the SBF-soaked and irradiated 10TiO_2_–Alg composite (Figure 3b).

**Figure 4:**
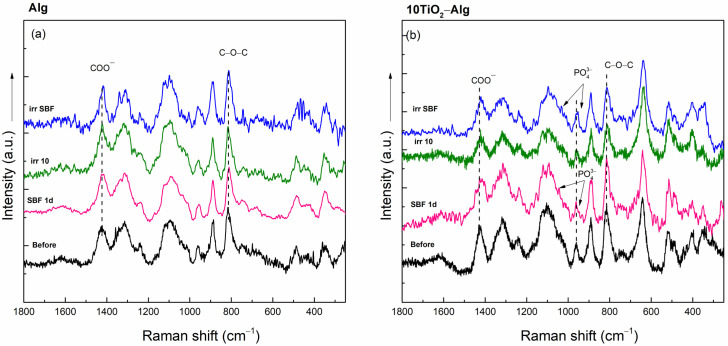
Raman spectra of the Alg (**a**) and 10TiO_2_–Alg (**b**) composites before treatment, after immersion in SBF for 1 day (SBF 1d), and after solar irradiation of the dry (irr 10) and SBF-soaked samples (irr SBF) for 10 min.

The Raman spectra of the Alg and 10TiO_2_–Alg hydrogel composites after immersion in SBF and after the solar irradiation of the dried and SBF-soaked samples presented small shifts of the bands at 817 and 1425 cm^−1^ assigned to the carboxylic group, indicating a structural modification (Figure 4). In addition, in the spectra of the 10TiO_2_–Alg composites, which were in contact with SBF, two other bands were present at 960 and 1040 cm^−1^, which can be assigned to P-O symmetric stretching vibrations, indicating the presence of apatite on the sample’s surface [27,28].

**Figure 5:**
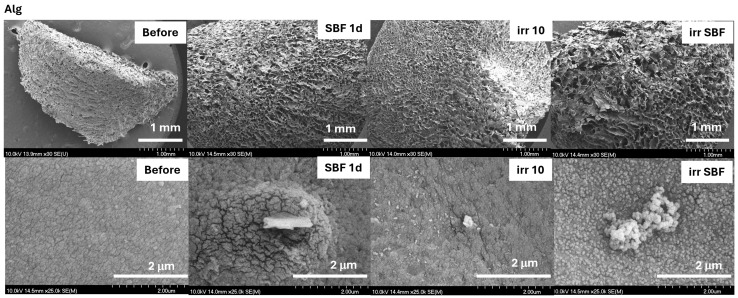
SEM micrographs of the Alg composites before treatment, after immersion in SBF for 1 day (SBF 1d), and after the solar irradiation of the dry (irr 10) and SBF-soaked samples (irr SBF) for 10 min. Scale bars: 1 mm (**first line**) and 2 μm (**second line**).

**Figure 6:**
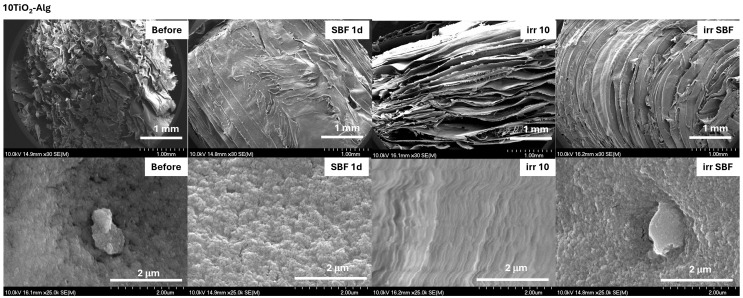
SEM micrographs of the 10TiO_2_–Alg composites before treatment, after immersion in SBF for 1 day (SBF 1d), and after the solar irradiation of the dry (irr 10) and SBF-soaked samples (irr SBF) for 10 min. Scale bars: 1 mm (**first line**) and 2 μm (**second line**).

The SEM micrograph confirmed the obtained spectroscopic results (Figure 5 and Figure 6). The SBF-immersed hydrogel composites showed a typical apatite layer on the surface with small apatite nanorods [29,30]. The formed apatite was more accentuated when the sample contained TiO_2_. Similar results were also obtained by Mallakpour et al. [31] and Kolathupalayam et al. [32], namely, the presence of TiO_2_ in the nanocomposites led to the formation of a higher amount of apatite on the samples’ surface. The surface of the solar-irradiated composite with TiO_2_ became more wrinkled, suggesting composite instability under solar irradiation. These changes were also visible in the SBF-soaked and irradiated 10TiO_2_–Alg composite surface (Figure 6).

### 2.3. TiO_2_–Chitosan–Alginate Composites

#### 2.3.1. Cross-Linking of the TiO_2_–Chitosan–Alginate Composites

**Figure 7:**
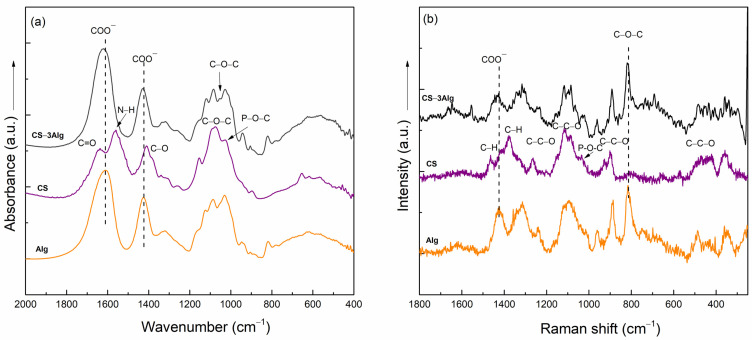
FT-IR (**a**) and Raman (**b**) spectra of the Alg, CS, and CS–3Alg composites.

We attempted to prove the cross-linking of the constituent materials by investigating the XRD patterns, FT-IR, and Raman spectra (Figure S7 and Figure 7) of the hydrogel composites as compared to those of the individual components, Alg and CS.

The XRD results displayed the amorphous nature of the CS–3Alg sample (Figure S7a). By adding TiO_2_ in the CS–3Alg system, the typical reflection at 2θ = 25.2° of TiO_2_ could be observed (Figure S7b). This was only visible in the case of the 10TiO_2_–CS–3Alg composite compared to the 10TiO_2_–Alg composite. We assumed that the Ti^4+^ cation coordinated with the alginate COO^−^ group. By adding CS into the matrix, too, competitive coordination took place, where the interaction of the COO− group with the OH− group of CS was much more favorable than with TiO_2_, and therefore, the TiO_2_ reflection did not change.

The FT-IR spectrum of the CS exhibited typical absorptions: amide I, amide II, and amide III bands (C=O, N-H, and C-N) at 1640, 1550, and 1320 cm^−1^, respectively, as well as C-O stretching vibrations at 1420, 1030, and 1090 cm^−1^, C-O-C bridge vibrations at 1150 cm^−1^, and O=C-N vibrations at 660 cm^−1^ (Figure 7a) [33,34]. The presence of β-glycerophosphate disodium salt hydrate (BGP) was evidenced by the shoulder at 1028 cm^−1^, assigned to aliphatic P-O-C stretching, and by the change in the intensity ratio of the amide I and amide II bands, indicating the occurrence of the chemical bond between BGP and CS [35]. The cross-linking between Alg and CS was evidenced by the shift of the vibration bands of the COO^−^ groups from 1610 to 1623 cm^−1^ and from 1425 to 1432 cm^−1^, as well as by the appearance of a new signal at 1055 cm^−1^. This band could be attributed to the vibration of the new C-O-C bond formed between Alg and CS. In this scenario, an ester group could be formed between the two polysaccharides via the esterification of the COO^−^ group of Alg and the OH group from CS. Another scenario could be that amide groups were formed between the COO^−^ group of Alg and the -NH_2_ group of CS. The second scenario could be confirmed by the disappearance of the band due to the -NH group vibration, located at 1550 cm^−1^ in the composite spectra. The Raman spectrum of CS showed typical bands at 471 cm^−1^, assigned to the C-C-O bending vibration, and at 895 and 1114 cm^−1^, assigned to the C-O-C stretching mode (Figure 7b) [36]. The Raman spectrum of CS–3Alg confirmed the FT-IR results; the bands assigned to the stretching vibrations of the C-O-C and COO- groups observed in the spectrum of alginate at 815 and 1425 cm^−1^ shifted to 821 and 1435 cm^−1^, respectively, indicating the formation of a chemical bond between Alg and CS.

**Figure 8:**
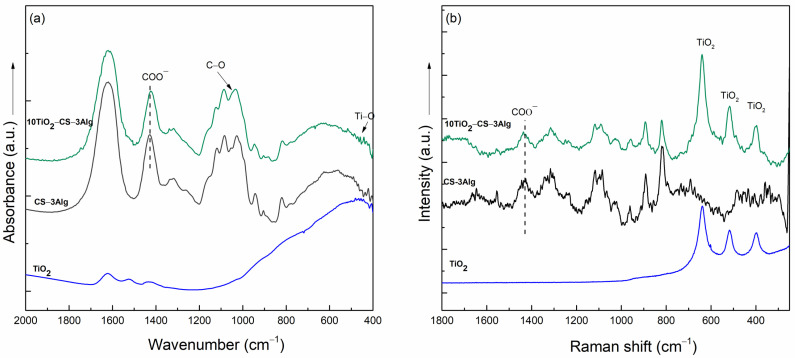
FT-IR (**a**) and Raman (**b**) spectra of TiO_2_, CS–3Alg, and the 10TiO_2_–CS–3Alg composites.

The cross-linking of TiO_2_ in the hydrogel composite was proven via IR spectroscopy by the shift of the band assigned to the vibration of the COO^−^ groups from 1432 to 1421 cm^−1^ as well as by the more pronounced band at 1050 cm^−1^, which could be assigned to the vibration of the C-O groups (Figure 8a). We assumed that the Ti^4+^ cation coordinated with the carboxyl group of Alg and the hydroxyl group of CS for better cross-linking between the two polysaccharides. The Raman spectrum showed similar results: the stretching vibration of the COO^−^ groups at 1432 shifted to 1439 cm^−1^, indicating the existence of a bond between TiO_2_ and the polymers (Figure 8b). The signals due to the TiO_2_ vibrations, which appeared in the spectrum of the blank sample at the values of 637, 516, and 395 cm^−1^, could also be observed in the spectrum of the composite.

**Figure 9:**
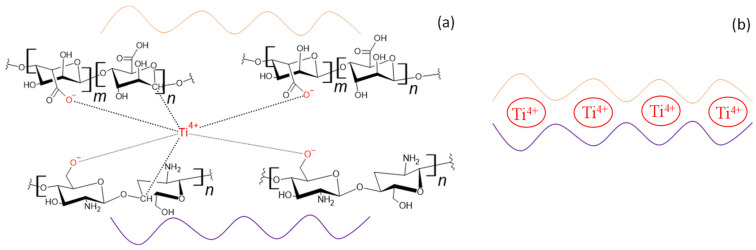
The schematical illustration of the coordination of TiO_2_ with the carboxyl group of alginate and the hydroxyl group of chitosan: (**a**) the structural perspective and (**b**) the overall coordination.

The schematical illustration of the coordination of Ti^4+^ with the carboxyl group of Alg and the hydroxyl group of CS can be seen in Figure 9. The hydroxyl group of the alginates could also interact with the metal center [37], but we assumed that with a carboxyl group, a much larger combination could be obtained according to the coordination shown in Figure 9b.

#### 2.3.2. In Vitro Assays of the TiO_2_–Chitosan–Alginate Composites

**Figure 10:**
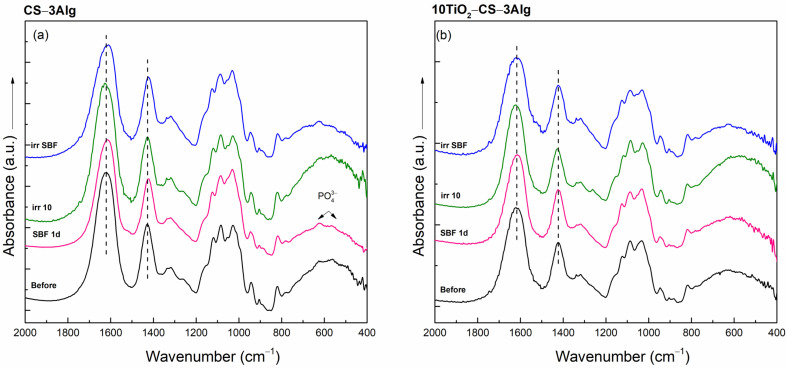
FT-IR spectra of the CS–3Alg (**a**) and 10TiO_2_–CS–3Alg (**b**) composites before treatment, after immersion in SBF for 1 day (SBF 1d), and after the solar irradiation of the dry (irr 10) and SBF-soaked samples (irr SBF) for 10 min.

The in vitro behavior of the hydrogel composites under different environmental conditions was further evaluated by analyzing the effect of their immersion in SBF and their possible damage by solar irradiation. All XRD patterns remained amorphous, and only the CS–3Alg composite after solar irradiation exhibited small changes in the structure that involved shifts of the reflection maxima to lower values (Figure S7). The FT-IR spectra of the solar-irradiated composites also showed changes, evidenced in the spectral domain between 800 and 400 cm^−1^ that became more pronounced (Figure 10a). These changes also appeared in the spectrum of the composite with TiO_2_ content, suggesting the appearance of structural changes under solar irradiation (Figure 10b).

**Figure 11:**
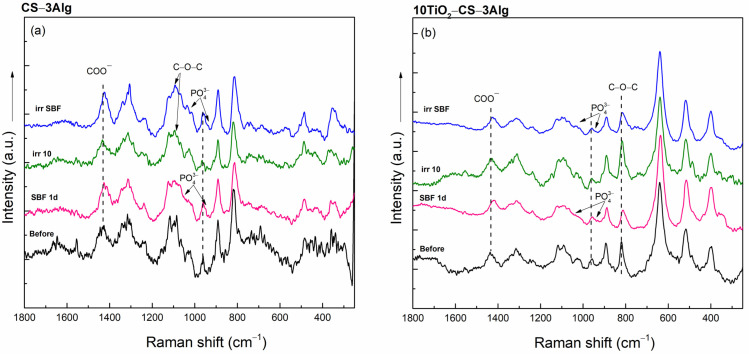
Raman spectra of the CS–3Alg (**a**) and 10TiO_2_–CS–3Alg (**b**) composites before treatment, after immersion in SBF for 1 day (SBF 1d), and after the solar irradiation of the dry (irr 10) and SBF-soaked samples (irr SBF) for 10 min.

The changes were more visible in the Raman spectra of CS–3Alg: in both irradiated spectra, the band at 1090 cm^−1^ assigned to the C-O-C stretching became more visible (Figure 11a). After SBF immersion, both composites presented the bands assigned to the P-O symmetric stretching vibrations at 960 and 1040 cm^−1^, as well as the band at 1435 cm^−1^, attributed to the carboxylic group that shifted to lower wavenumbers (Figure 11). This suggests that an apatite layer started to grow on the surface after SBF contact.

**Figure 12:**
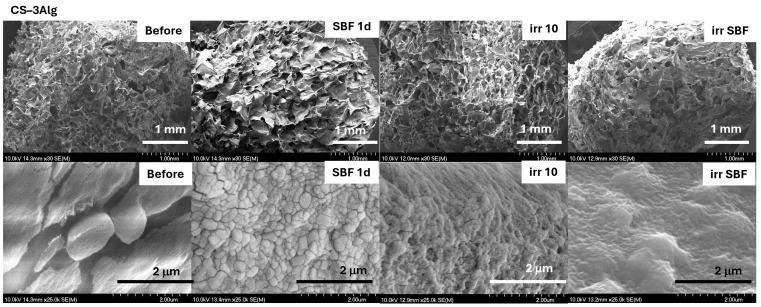
SEM micrographs of the CS–3Alg composites before treatment, after immersion in SBF for 1 day (SBF 1d), and after the solar irradiation of the dry (irr 10) and SBF-soaked samples (irr SBF) for 10 min. Scale bars: 1 mm (**first line**) and 2 μm (**second line**).

**Figure 13:**
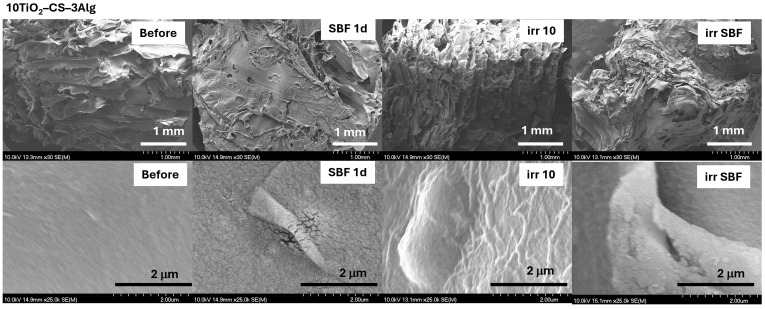
SEM micrographs of the 10TiO_2_–CS–3Alg composites before treatment, after immersion in SBF for 1 day (SBF 1d), and after the solar irradiation of the dry (irr 10) and SBF-soaked samples (irr SBF) for 10 min. Scale bars: 1 mm (**first line**) and 2 μm (**second line**).

The surfaces of all hydrogel composites immersed in SBF showed a typical morphology of an apatite layer (Figure 12 and Figure 13). After solar irradiation, the composite with TiO_2_ content presented a bit of a wrinkled surface but appeared less affected than the TiO_2_–Alg composite (Figure 13). This indicates that, by adding CS into the TiO_2_–Alg composite, it became more stable under solar irradiation.

### 2.4. Porosity, Biodegradation, and Swelling Ratio of the Composites

Highly porous composites were obtained for all the studied materials. However, small differences appeared. In the case of Alg, a porosity of 89% was obtained, and by adding TiO_2_, the porosity decreased to 69%. The same trends could be observed in the case of the CS–3Alg composites. For the CS–3Alg composite, a porosity of 96% was obtained, and by adding TiO_2_, the porosity decreased to 82%. It should be mentioned that the CS presence slightly increased the porosity.

**Figure 14:**
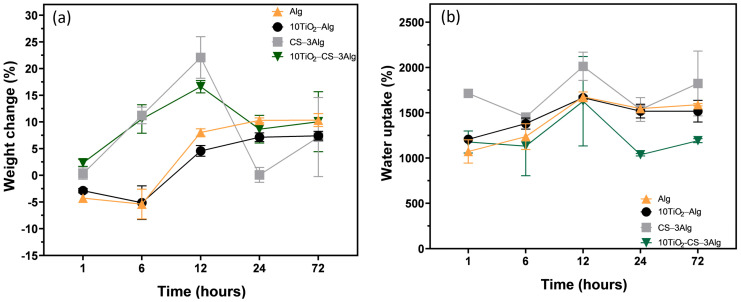
Biodegradation (**a**) and swelling ratio (**b**) of the Alg, 10TiO_2_–Alg, CS–3Alg, and 10TiO_2_–CS–3Alg hydrogels.

The biodegradation and water absorption capabilities of the samples were followed for three days, considering that any wound dressing must be changed within a maximum of three days. As expected, the biodegradation in the first hour was much faster in the samples with CS content (Figure 14a). After 72 h, the difference was minimal. As seen after immersion in SBF, an apatitic layer appeared on the surface of the samples, which we also saw through the increase and decrease in the weight. The water absorption capacities of the hydrogels were not negatively influenced by the TiO_2_ content (Figure 14b). In the case of the CS–3Alg composite, it shrunk slightly but still had an excellent swelling ratio.

### 2.5. Cell Viability Evaluation of the Composites

**Figure 15:**
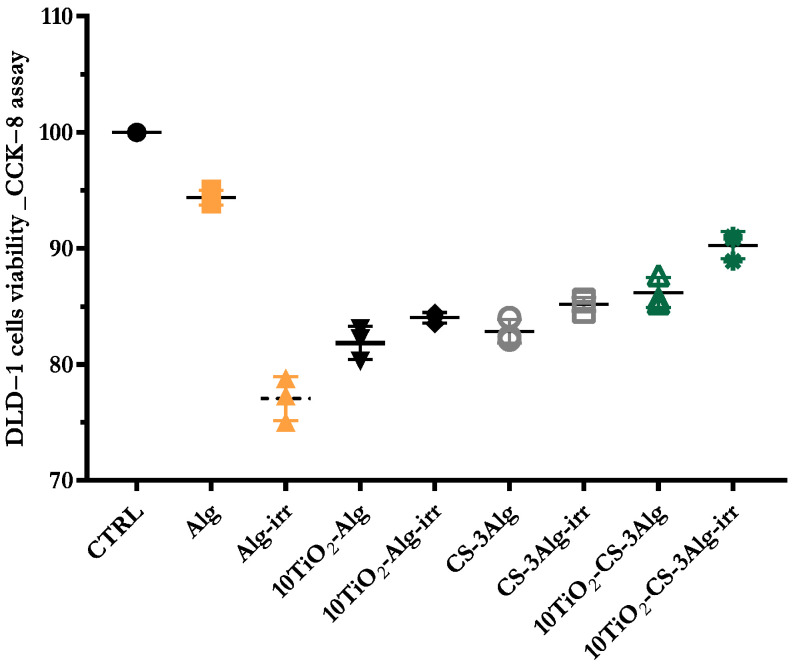
HaCaT cell viability after 24h of interaction with the Alg, 10TiO_2_–Alg, CS–Alg, and 10TiO_2_–CS–3Alg composites before and after solar irradiation (CTRL: control).

The viability of HaCaT cells after 24 h of incubation with the Alg, 10TiO_2_–Alg, CS–3Alg, and 10TiO_2_–CS–3Alg hydrogel composites was evaluated using the CCK-8 assay (Figure 15). The results of cell viability indicated a certain inhibition of cell proliferation dependent on the type of composite and, respectively, on the preceding treatment. For the irradiated composites, the inhibition of cell proliferation could be observed. Specifically, for the irradiated Alg composite, the average cell viability was 77.05% ± 1.54 compared to the non-irradiated Alg, where the average viability was 94.38% ± 0.52. However, the trend was not similar in the 10TiO_2_–Alg hydrogel composite, where the average cell viability before irradiation was 81.85% ± 1.16, and after irradiation, it was 84.02% ± 0.37; the differences were not statistically significant. Similarly, the viability of HaCaT cells in the presence of the non-irradiated CS–3Alg sample was 82.84% ± 0.84 compared to the irradiated composite, where the average cell viability showed a slight increase, 85.19% ± 0.47. For the 10TiO_2_–CS–3Alg hydrogel composite, the average cell viability before irradiation was 86.18% ± 1.06 compared to the irradiated probe, where the average cell viability showed a moderate increase, 90.26% ± 0.96. In conclusion, none of the composites presented toxicity, and by adding TiO_2_ in the composites, the cell viability of the solar-irradiated samples slightly increased, suggesting the solar protecting effect of TiO_2_.

## 3. Conclusions

Throughout this paper, we successfully prepared and characterized hydrogel composites with Alg, CS, and TiO_2_ and investigated the behavior of TiO_2_ in a body-simulated medium irradiated with solar-spectrum light. The samples were successfully cross-linked, despite the water-insolubility of CS and the challenging homogeneity of all the materials.

After the in vitro tests, the Raman spectra and SEM images showed that the surface of the immersed composites changed, and a thin apatite-like layer covered the surface of the samples. This could demonstrate the medical properties of the hydrogel composites and that a biological fluid did not degrade them but supported and created a layer on the surface.

With regard to UV stability, it was shown that the samples in which CS (CS–Alg) was added were more stable in the irradiated environment than the samples without it (Alg). By adding anatase to the polymeric composites, the UV stability increased, as proven by the better keratinocyte cell viability after 24 h. However, in order to clarify possible inflammatory effects and neurotoxicity, further tests are needed.

## 4. Materials and Methods

### 4.1. Materials

All chemicals were used as received without further purification. Ultrapure water and absolute ethanol were used throughout the experiments. For the synthesis of TiO_2,_ tetraisopropylorthotitanate (TTIP, >98%, Merck, Darmstadt, Germany) ethanol (absolute, Chimreactiv, Bucharest, Romania) and glacial acetic AcOH, Chempur, Karlsruhe, Germany) were used. For the synthesis of the composites, sodium alginate (CAS 9005-38-5) and chitosan (medium molecular weight) polymeric powder was used from Sigma-Aldrich. β-glycerophosphate disodium salt hydrate (BGP) was acquired from Sigma-Aldrich, and calcium chloride (CaCl_2_, >97%) was acquired from Penta. For the preparation of the simulated body fluid (SBF), sodium chloride (NaCl, 99.9%, Poch Basic, Gliwice, Poland), sodium hydrogen carbonate (NaHCO_3_, Penta, Singapore), potassium chloride (KCl, Penta), dipotassium hydrogen phosphate (K_2_HPO_4_, 99%, Penta), magnesium chloride hexahydrate (MgCl_2_∙6H_2_O, 99%, Merck), calcium chloride (CaCl_2_, >97% Penta), sodium sulfate (Na_2_SO_4_, 99%, Nordic Chemicals, Cluj-Napoca, Romania), tris(hydroxymethyl)aminomethane (TRIS, 99.8%, Merck), and hydrogen chloride (HCl, 1N Nordic Chemicals) were used.

### 4.2. Composite Synthesis

#### 4.2.1. Synthesis of the TiO_2_ Particles

TiO_2_ was synthesized via a modified sol–gel hydrothermal method. A total of 17.5 mL of TTIP was added into 60 mL of absolute EtOH under vigorous stirring. To this first solution, an aqueous acetic acid solution (85% *w*/*w*) was added dropwise under constant agitation at room temperature. The total molar ratio of the precursors of TTIP: EtOH: AcOH: H_2_O was 1:17.1:1.74:1. The obtained synthesis solution was immediately transferred into a stainless-steel autoclave with a Teflon lining and treated at 180 °C for 1 h. The autoclave was cooled down naturally to room temperature after the hydrothermal treatment. The gained material was centrifuged and washed at least 3 times with distilled water until the obtained suspension of the particles had a pH of 7, after which it was dried for 12 h at 80 °C.

#### 4.2.2. Synthesis of the Composites

TiO_2_–Alginate composites: Polymeric solutions of 4% (*w*/*v*) sodium alginate were dissolved at 80 °C in ultrapure water under continuous stirring to obtain the composites. To obtain a uniform particle size, TiO_2_ was ultrasonicated and then added to the polymeric solution in a weight ratio of 10% (10TiO_2_–Alg). For reference, a pure alginate (Alg) sample was also prepared.

TiO_2_–Chitosan–Alginate composites: Component A: The hydrogel composites were prepared, starting from 1% (*w*/*v*) chitosan dissolved in glacial acetic acid under constant stirring at room temperature for 24h. The gelling agent solution was prepared by dissolving BGP in ultrapure water at a concentration of 1.8 g∙L^−1^. The chitosan and BGP solutions were mixed in an ice bath in a ratio of 31.5:1. The pH value of the solution was increased to 6. Component B: Sodium alginate (4% *w*/*v*) was dissolved at 80 °C in ultrapure water under continuous stirring. To obtain a uniform particle size, TiO_2_ was ultrasonicated and then added to the alginate solution. Component B was added to component A using a syringe. The weight ratio of chitosan and Alg was 1:3 (CS–3Alg), and TiO_2_ was added in a weight ratio of 10% to the polymeric solution (10TiO_2_–Alg). For reference, chitosan with BGP was also prepared (CS).

All the composites, including the TiO_2_–alginate and TiO_2_–chitosan–alginate composites, were cast in a 96-well plate, kept frozen at −18 °C for 24 h and lyophilized for 24 h in a vacuum freezer dryer (BK-FD series, Biobase Bioindustry, Shandong Co., Ltd., Jinan, Shandong, China). The composites were cross-linked in a 4% CaCl_2_ solution for 4 h. After cross-linking with calcium ions, the hydrogel composites were washed with ultrapure water, and the lyophilization process was repeated.

### 4.3. Investigation Methods

X-ray Diffraction (XRD): The XRD measurements were performed using a Shimadzu XRD 6000 diffractometer (Kyoto, Japan), which was operated with CuKα radiation (λ = 1.54 Å) and a Ni filter. The diffraction patterns were recorded in the 2θ range of 10–80° with a scan speed of 2°/min. The crystalline degree of the TiO_2_ sample was calculated using FullProofSuite software (V 4.1) with the following equation:

(1) $$Degree\;of\;crystallinity\left(\%\right)=\frac{I_{crystalline}\times 100}{I_{total}}$$

I_crystalline_ and I_total_ represented the integrated intensities of the total crystalline reflection of TiO_2_. All the reflection was considered.

Transmission Electron Microscopy (TEM): The TEM images were recorded using an FEI Technai G2 F20 high-resolution TEM (Hillsboro, ON, USA) equipped with a 200 kV, W cathode. The samples were suspended in H_2_O and dropped on a 300-mesh Cu grid. The obtained images were interpreted with ImageJ software.

Fourier transform Infrared Spectroscopy (FT-IR): The FT-IR absorption spectra were recorded at room temperature in the reflection configuration with a Jasco FT-IR 6600 (Jasco, Tokyo, Japan) spectrometer, using the well-known KBr pellet technique with the following parameters: 400–4000 cm^−1^ spectral range, 4 cm^−1^ spectral resolution.

Raman Spectroscopy: The Raman spectra were acquired using a multilaser confocal Renishaw InVia Reflex Raman spectrometer equipped with a 1200 lines/mm grating. The samples were excited with the 785 nm laser line at maximal laser power (300 mW). The Raman spectra were recorded in the 100–2000 cm^−1^ spectral range with an acquisition time ranging from 10 to 30 s using a 100× (NA 0.9) objective.

Scanning Electron Microscopy (SEM): The SEM micrographs were recorded with a Hitachi S-4700 Type II cold field emission scanning microscope (Tokyo, Japan) operated at an acceleration voltage of 10 kV. The magnification of the millimeter-scale SEM micrographs was 30×, and the magnification of the micrometer-scale images was 25×.

Ultraviolet–Visible spectroscopy (UV–Vis): the UV–Vis absorption spectra were recorded using a Jasco V780 UV–VIS spectrophotometer (Jasco, Tokyo, Japan) with a spectral resolution of 0.5 nm and a scan speed of 100 nm/min.

Solar Simulator: The irradiation of the samples was carried out with a 500W Fully Reflective Solar Simulator, SS Series from Sciencetech (London, OH, USA) using an Am 1.5G filter and a working distance of 9 cm.

### 4.4. Porosity, Biodegradation, and Swelling Ratio Measurements

The composite porosity was measured using the liquid displacement method [38]. Ethanol was used as the displacement liquid, and the percentage of porosity was calculated according to the following formula:

(2) $$P\left(\%\right)=\left[\frac{W_{1}-W_{0}}{\rho_{EtOH}\times V_{0}}\right]\times 100$$

where W_0_ is the dry weight of the composite, W_1_ is the weight of the composite saturated with ethanol, ρ_EtOH_ is the density of the ethanol, and V_0_ is the initial volume of the composite scaffold.

The biodegradation and swelling ratio assays were performed in simulated body fluid, SBF. The SBF was prepared according to Kokubo’s protocol [16,17]. The solution was buffered at pH 7.4 at 37 °C. After the incubation, the samples were rinsed with distilled water, their surface was wiped, and their wet weights (W_w_) were measured. Then, they were dried at 37 °C for 48 h to obtain their constant weight, then, they were weighed to obtain their dry weights (W_d_). The weight loss was calculated as a percentage according to the following formula:

(3) $$Weight\;loss\%=\left[\frac{Wi-Wd}{Wi}\right]\times 100$$

where W_i_ is the initial weight of each sample.

The water absorption was calculated as a percentage using the following formula:

(4) $$Water\;absorption\%=\left[\frac{Ww-Wd}{Wd}\right]\times 100$$

All measurements were repeated three times.

### 4.5. In Vitro Assays

#### 4.5.1. In Vitro Bioactivity Assay

In vitro bioactivity tests were carried out in SBF by immersing the samples for 24 h at 37 °C. The surface of the composite per volume of SBF was 40 mm^2^·mL^−1^. After incubation, the samples were rinsed with distilled water and dried at 37 °C for 48 h.

#### 4.5.2. In Vitro Stability under Sunlight Irradiation

The stability of the composites under sunlight was tested in two ways: (1) the dry composites were exposed to simulated solar irradiation for 10 min, and (2) the composite hydrogels were immersed in SBF for 30 min and were exposed to simulated solar irradiation for 10 min.

#### 4.5.3. In Vitro Cell Viability Assay

The study investigated the proliferative effects of the 10TiO_2_–Alg and TiO_2_–CS–3Alg hydrogel composites on the HaCaT (human epidermal keratinocyte line) cell line using the CCK-8 assay. The fundamental principle underlying the CCK-8 assay involves the enzymatic action of cellular dehydrogenase enzymes present in metabolically active cells, which facilitate the reduction of a water-soluble tetrazolium salt, WST-8, into a soluble formazan compound within the cell culture medium. The amount of formazan produced is directly proportional to the number of living cells in the culture. HaCaT cells (1x105 cells/well/24-well plates, CytoOne, Cell Culture, Minneapolis, MN, USA) were cultured in DMEM medium (Gibco Life Technologies, Paisley, UK) supplemented with 10% fetal bovine serum (Sigma-Aldrich, St. Louis, MO, USA), 2 mM L-Glutamine (Sigma-Aldrich, St. Louis, MO, USA), and 1% anti-biotics-anti-mycotics (Gibco Life Technologies, Paisley, UK) under standard conditions of 37 °C, 5% CO_2_, and 60% humidity. After 24 h, the composites before and after irradiation were introduced into the inserts, while wells without inserts and composites served as the controls. Following another 24 h of incubation, the inserts were removed, and the CCK-8 solution (Sigma-Aldrich, St. Louis, MO, USA) was added to each well, followed by further incubation for 4 h at 37 °C in the dark. Subsequently, the absorbance of each well was quantified at 450 nm using a microplate reader (Bio-Rad, Hercules, CA, USA). All experiments were conducted in triplicates, and the data were presented as the mean  ±  SD. Cell survival (%) was calculated based on the optical densities normalized to the control.

## Data Availability

All data and materials are available upon request from the corresponding author. The data are not publicly available due to ongoing research using a part of the data.

## Supplementary Materials

The following supporting information can be downloaded at: https://www.mdpi.com/article/10.3390/gels10060358/s1, Figure S1: XRD pattern of TiO_2_ (a), TEM image of TiO_2_ nanoparticles (b), and the size distribution histogram for the sample (c); Figure S2: FT-IR (a) and Raman (b) spectrum of TiO_2_, Figure S3: UV-Vis (a) and first derivate (b) spectrum of TiO_2_, Figure S4: XRD for TiO_2_ before, after immersion in SBF for 1 day, after solar irradiation for 10 min, and after solar irradiation in SBF for 10 min, Figure S5: First derivate spectra deconvolution of Alginate (a) and 10TiO_2_–Alg (b) composites, Figure S6: XRD pattern of Alg (a) and 10TiO_2_–Alg (b) composites before, after immersion in SBF for 1 day, after solar irradiation for 10 min, and after solar irradiation in SBF for 10 min, Figure S7: XRD pattern of CS–3Alg (a) and 10TiO_2_–CS–3Alg (b) composites before, after immersion in SBF for 1 day, after solar irradiation for 10 min, and after solar irradiation in SBF for 10 min.
