# Supplementary material for: TiO2–Alginate–Chitosan-Based Composites for Skin Tissue Engineering Applications
Source: Gels. 2024 May 22;10(6):358. doi: 10.3390/gels10060358 (PMC11203036; doi:10.3390/gels10060358)
Supplement: Supplementary file 1 [file gels-10-00358-s001.zip › gels-2983075-supplementary.pdf]

Supplementary Information

TiO_2 –alginate-chitosan-based composites for skin tissue engineering applications

1. TiO_2 particle characteristics

The XRD patterns of TiO_2 show reflections for the anatase crystalline phase (Fig. S1a.; COD 00-101-0942). The TiO_2 particles are spherical and polydisperse with an average size of 1.82 μm. This resulted from TEM images (Fig. S1b).

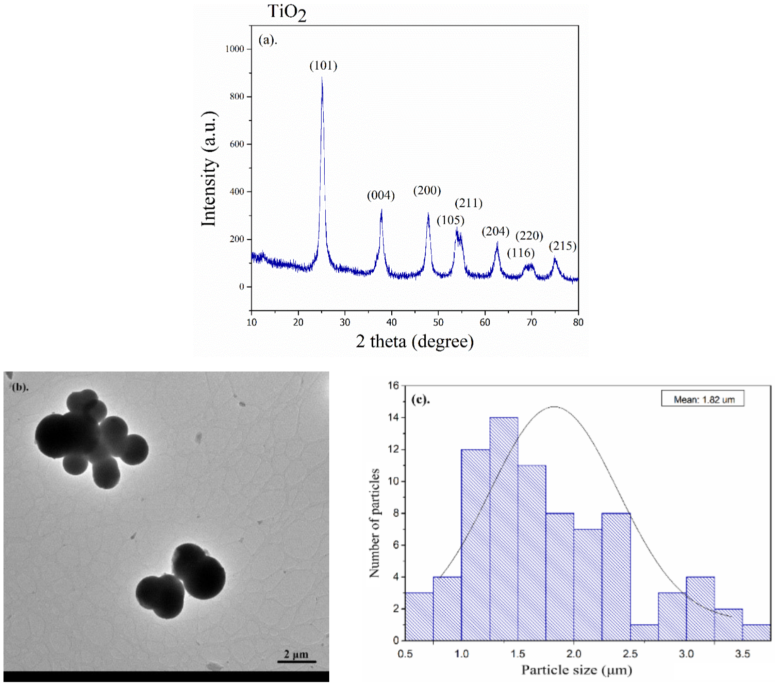

Figure S1. XRD pattern of TiO_2 (a), TEM image of TiO_2 nanoparticles (b), and the size distribution histogram for the sample (c)

The typical bands assigned to TiO_2 vibrations can be observed in the FT-IR and Raman spectra of TiO_2 illustrated in Figures S2a and S2b. The Ti-O-Ti and Ti-O stretching modes give rise to the absorption signals from 721 and 463 cm^{-1}, which are specific to the anatase titania. On the other hand, the Raman spectrum shows bands at 142, 395, 518, and 638 cm^{-1} characteristics to anatase titania, confirming thus the results revealed from FT-IR spectrum analysis.

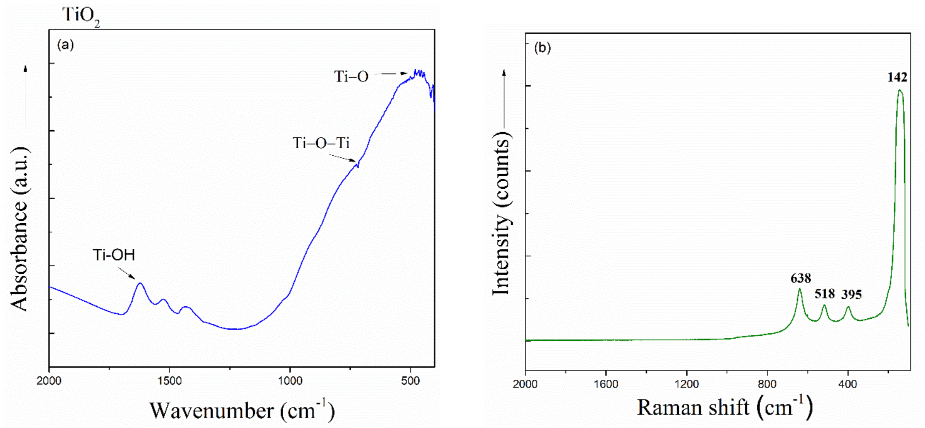

Figure S2. FT-IR (a) and Raman (b) spectrum of TiO_2

The UV-Vis spectrum of TiO_2 (Figure S3a) shows the typical absorption threshold of the anatase crystalline phase. To completely exclude the appearance of the rutile crystalline phase, we have applied the first derivative on the UV-Vis spectrum of the sample (Fig. S3b), and only one minimum was observed, at 374 nm (3.31 eV). According to our previous study, where composites with different amounts of anatase and rutile phases were investigated, we can conclude that if a small amount of rutile crystalline phase is present in the sample, it would be visible in the first derivative spectrum. Thereby, we conclude that no rutile crystalline phase is present in the TiO_2 nanoparticles structure.

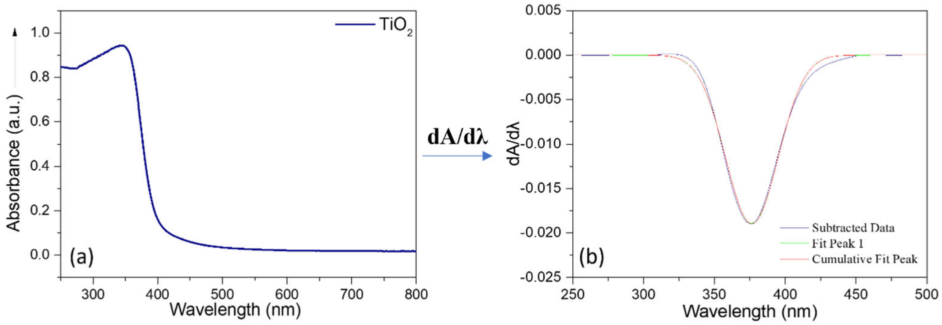

Figure S3. UV-Vis (a) and first derivate (b) spectrum of TiO_2

2. In vitro assays of TiO_2 particles

The XRD diffractograms of TiO_2 (Figure S4) are very similar and show the crystallinity of the TiO_2, with the same crystalline planes.

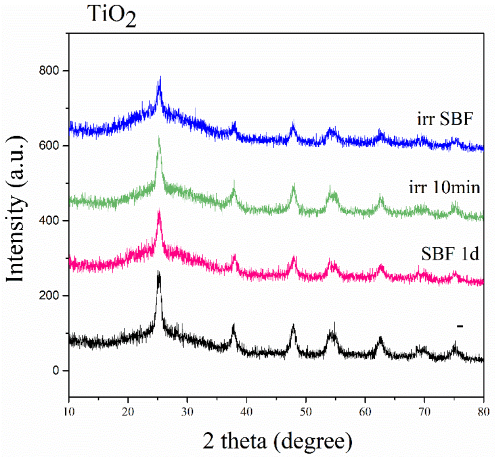

Figure S4. XRD for TiO_2 before, after immersion in SBF for 1 day, after solar irradiation for 10 min, and after solar irradiation in SBF for 10 min

3. Cross-linking of TiO_2-alginate composites

First derivate spectra deconvolution of Alginate (Figure S5a) and 10TiO_2-Alg (Figure S5b). We notice two different minimum values, one corresponding to alginate (324 nm), and the other one corresponding to anatase (367 nm).

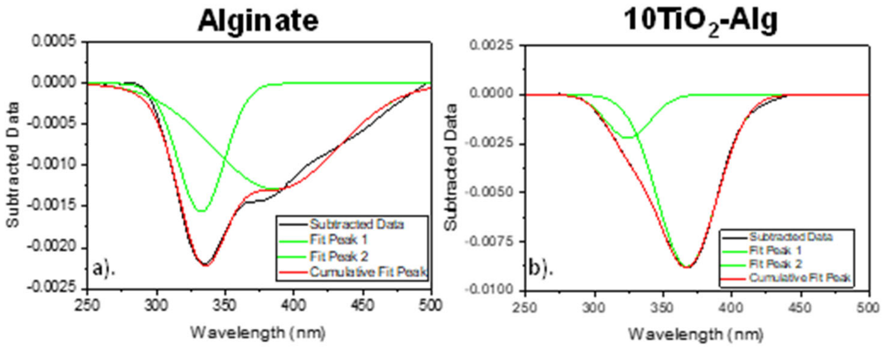

Figure S5. First derivate spectra deconvolution of Alginate (a) and 10TiO_2-Alg (b) composites

4. In vitro assays of TiO_2-alginate and TiO_2-chitosan-alginate composites

The diffractograms of Alg and 10TiO_2-Alg composites after immersion in SBF for 1 day, as well as those recorded after solar irradiation of the dried samples and of the SBF-soaked samples for 10 min, are very similar, the amorphous character of the samples being shown (Figure S6 a & b).

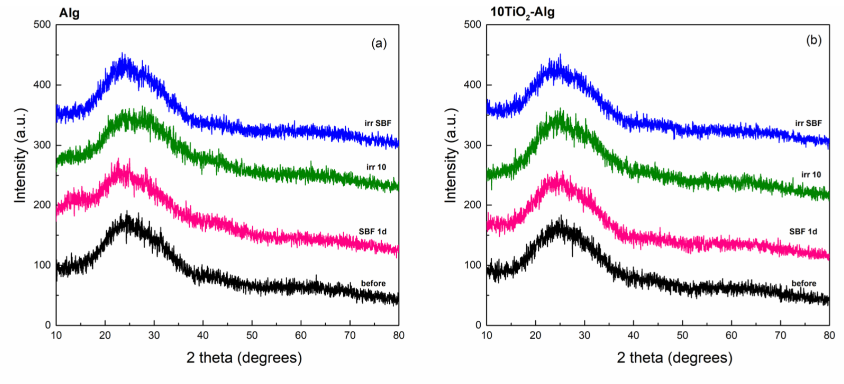

Figure S6. XRD pattern of Alg (a) and $10TiO_2$-Alg (b) composites before, after immersion in SBF for 1 day, after solar irradiation for 10 min, and after solar irradiation in SBF for 10 min

The XRD diffractograms of CS-3Alg (Figure S7a) show the amorphous nature of the sample. By adding TiO_2 in the CS-3Alg system, the typical reflection at $2\theta=25.2°$ of TiO_2 is observed (Figure S7b).

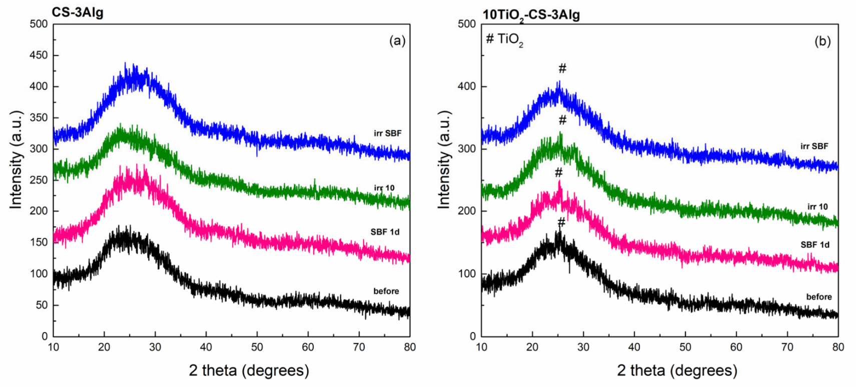

Figure S7. XRD pattern of CS-3Alg (a) and $10TiO_2$-CS-3Alg (b) composites before, after immersion in SBF for 1 day, after solar irradiation for 10 min, and after solar irradiation in SBF for 10 min
